# A Passive Monitoring System in Assisted Living Facilities: 12-Month Comparative Study

**DOI:** 10.3109/02703181.2011.650298

**Published:** 2012-02-16

**Authors:** Pankaj A Patel, Candace Gunnarsson

**Affiliations:** 1 Senior Director, Health Economics, GE Healthcare, Barrington, IL 60010, USA; 2 President, S^2^ Statistical Solutions, Inc., Cincinnati, OH 45241, USA

**Keywords:** passive monitoring, assisted living, falls, hospitalizations

## Abstract

The GE QuietCare® passive monitoring system uses advanced motion sensor technology that learns the daily living patterns of senior community residents and sends alerts when certain out-of-the-ordinary events occur. This study compared falls, hospitalizations, care level changes, and resident attrition between two similar assisted living facilities where one facility adopted the QuietCare® monitoring system and the other did not over a 12-month period. Average falls per week were significantly lower in the QuietCare® facility than the control facility. There was also a trend toward fewer weekly hospitalizations in the QuietCare® facility. There was higher resident retention at the QuietCare® facility. This study provides evidence of direct benefits to both the resident and the facility for the use of QuietCare®. There was a significant reduction in the number of falls, as well as a general facility performance improvement measured by care level consistency and higher resident retention rates.

## INTRODUCTION

Demand for assisted living facilities is increasing as the baby-boom generation ages and the percentage of older Americans increases over the next 25 years. According to the US Census Bureau, there were over 38 million people aged 65 years and older living in the United States in 2010 and that number is growing (United States Census Bureau, 2010). While older adults typically prefer remaining in their homes, if this is no longer possible, a second choice might be in a home-like environment of an assisted living facility rather than moving to the more restrictive nursing home environment (Chapin & Dobbs-Kepper, 2001). One factor limiting the ability of seniors to stay in an assisted living facility for long-term is age-related functional decline, including a decline in the ability to perform activities of daily living, resulting in an increased need for assistance from caregivers (Aud, 2004; Hawes, Phillips, Rose, Holan, & Sherman, 2003; Kissam, Gifford, Mor, & Patry, 2003). Thus, while seniors may prefer to remain in the least restrictive environment possible, increasing dependence and functional decline often result in a need for increased care and transfer to a nursing home (Alexander et al., 2008).

Adverse events related to the mobility of seniors, including wandering and falls, are common. These adverse events can have a negative impact on patient health, functional status, and quality of life (Nelson et al., 2004). Over half of assisted living residents have some type of cognitive impairment (Alzheimer's Association, 2011). Wandering affects 39% of cognitively impaired nursing home residents and up to 70% of community-residing seniors with cognitive impairments (Nelson et al., 2004). Falls are a major concern for assisted living facilities. Studies show that 30 to 40% of seniors fall annually (Hausdorff, Rios, & Edelberg, 2001; Sattin, 1992; Tinetti, Speechley, & Ginter, 1988; Verghese, Ambrose, Lipton, & Wang, 2010) and those who fall are 2–3 times more likely to have repeat falls (Tinetti et al., 1988). More troubling is the fact that one-third of falls result in serious injuries (Fleming & Brayne, 2008). Unfortunately, only half of hospitalized residents return to assisted living facilities after a fall (Sattin et al., 1990).

In addition to the adverse effects of falls on seniors, assisted living facilities are also negatively affected. When a resident is hospitalized, the assisted living facility must hold the resident's spot in the facility; however, Medicare will not reimburse the facility during the hospitalization and therefore the financial toll to the facility can be great. Assisted living facilities charge residents more as they move from level 1 (basic) care to higher levels of care based on their ability to perform activities of daily living as greater monitoring is required on the part of the assisted living facility.

New technologies have been implemented in assisted living facilities to help prevent mobility-related adverse events, including door alarms and signal-transmitting devices (Nelson et al., 2004). Recently, remote monitoring systems that detect behavioral patterns and signal facility staff of unexpected behavior have been developed (Barger, Brown, & Alwan, 2005; Cardinaux, Brownsell, Hawley, & Bradley, 2008). These systems employ probabilistic modeling of behavior through association of attributes with each occurrence of a type of activity. Since behavior follows regular patterns, the models are able to use historical data to identify these patterns. Deviations from the model are considered abnormal (Cardinaux et al., 2008) and therefore can be used to alert of potential danger.

The GE QuietCare® passive monitoring system utilizes motion sensors to track resident activity in assisted living facilities. The sensors relay resident activity to a data communicator, which then transfers information to an off-site server where the data are analyzed via algorithms to identify significant changes in daily routine that may signal urgent situations or potential changes in behavior that could lead to an urgent situation. If a potentially urgent situation is identified, eldercare facility staff is notified so that they can respond promptly. Unlike rules-based systems, the QuietCare® algorithms learn the behavioral patterns of individual residents, leading to a personalized care plan for each resident (GE QuietCare, 2011).

The objective of this study was to compare falls, hospitalizations, care level changes, and resident attrition over a 12-month period between two similar assisted living facilities where one facility adopted the QuietCare® monitoring system and the other did not.

## METHODS

Data were collected at two similar assisted living facilities from January 2009 through February 2010. One facility adopted use of the GE QuietCare® monitoring system during the study period (QuietCare® facility), while the second did not (control facility). The assisted living facilities were geographically distinct to limit any contamination of care; however, the same company owned both, so there may be similar protocols in place. Data were compiled in a spreadsheet on a weekly basis and consisted of input from the GE QuietCare® database as well as manual entries from facility staff for data that included care levels, falls, and hospitalizations.

Due to uncertainties around the number of weeks included in baseline data prior to week 0, week 0 is excluded from the analysis for each of the two facilities. Means, medians, and percentages of all variables were compared between facilities using the Kruskal–Wallis nonparametric test of differences in means. In addition, the percentage of residents at care levels 1, 2, or 3 at the resident baseline and at final measurement (last week of study or when a resident left) was also analyzed.

A multiple regression model for total falls and total hospitalizations in each facility was developed. The variables used in the model were those significantly different in the bivariate analysis. Patient characteristics, including resident age, gender, and comorbidities, were included in the comparison of the two facilities. Multivariable analytics were utilized to compare the average number of falls and hospitalizations between the two facilities taking into account the difference in the number of residents, age, and fall risk. General trends for care level and patient attrition over time were evaluated.

A time series model for total falls and total hospitalizations was fit to the data (Tjøstheim & Paulsen 1983), using a lag of 4 weeks. Including the facility ID as a dichotomous variable, differences in outcomes between facilities were assessed. Model fit was assessed using residual and the Durbin–Watson analysis.

All data were imported and maintained in a SAS® data file. Tabulation of summary statistics, graphical presentations, and data analysis were performed using SAS® software, version 9.1.

## RESULTS

Over the length of the 12-month study period, the weekly average number of residents in the QuietCare® site was 39.4 and 42.4 living in the control site. The mean (standard deviation [SD]) age was similar in both cohorts (88.0 [5.4] for QuietCare®; 88.5 [5.3] for control). The average (SD) length of stay was similar for residents in the QuietCare® facility (27.2 [23.7] months) and in the control facility (26.7 [23.9] months). At baseline, 76.3% of the residents in the QuietCare® facility were at care level 1, 22.2% of the residents were at care level 2, and 2.6% were at care level 3. There were fewer residents at care level 1 (68.9%) in the control facility, while 26.7% were at care level 2 and 4.4% were at care level 3 (Table 1). There was higher resident retention at the QuietCare® facility over the 52-week study period. Sixty three percent of the baseline residents at the QuietCare® facility remained at the facility for the entire 12-month period versus 49% at the control facility.

**TABLE 1:** Baseline Resident Characteristics

	QuietCare® Facility	Control Facility
N	38	45
Mean (SD) Age (Years)	88.0 (5.4)	88.5 (5.3)
Mean (SD) Length of Stay (in Months)	27.2 (23.7)	26.7 (23.9)
Baseline Care Level (%)		
1.	76.3	68.9
2.	22.2	26.7
3.	2.6	4.4

**Figure 1:**
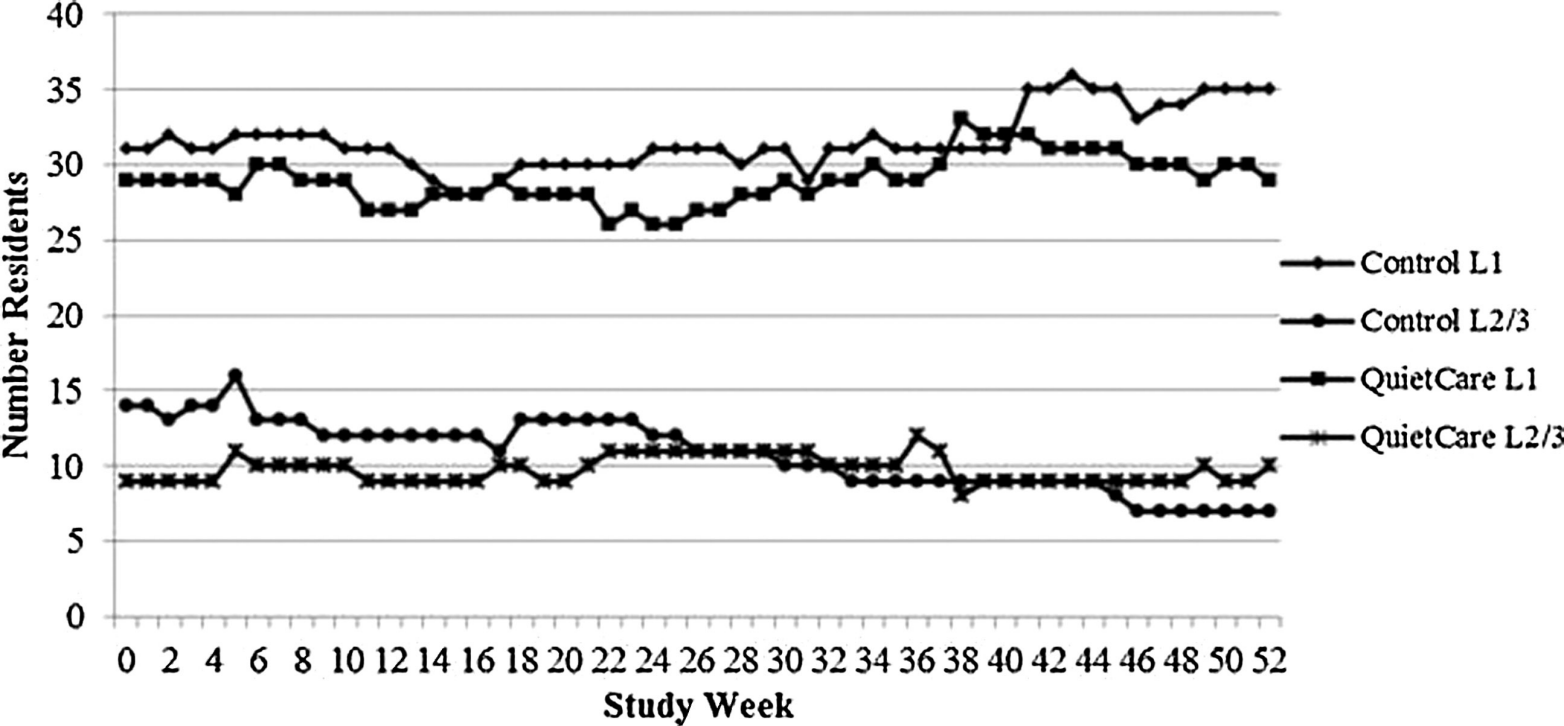
Care levels of residents through 52 weeks.

Over the 52-week study period, the QuietCare® facility maintained a consistent number of residents in care level 2 or 3, while at the control facility there was a downward trend in the number of residents in care level 2 or 3 (Figure 1). The percentage of residents in care levels 2 and 3 at the end of the study was higher at the QuietCare® facility versus the control facility (24% versus 17% respectively).

When looking at total falls over the 52-week study period, 25 occurred in the QuietCare® facility, while 69 occurred at the control facility. Average falls per week were significantly lower in the QuietCare® facility than the control facility (0.48 vs. 1.33; *p* = 0.0001). Total hospitalizations were also lower in the QuietCare® facility (48) than the control facility (57), resulting in a trend toward fewer weekly hospitalizations in the QuietCare® facility (0.92 versus 1.08); however, this difference did not reach statistical significance (Table 2).

**TABLE 2:** Falls and Hospitalizations

Events (Through Week 52)	QuietCare® Facility	Control Facility	*p*-value
Total Falls	25	69	
Average Falls per Week	0.48	1.33	0.0001
Total Hospitalizations	48	57	
Average Hospitalizations per Week	0.92	1.08	0.480

The results of the time series model for total falls and hospitalizations showed that both decreased over time at the two facilities. Weekly falls for the QuietCare® facility were, on average, 1.08 fewer (*p* = 0.008) than for the control facility after controlling for the secular trend. Similarly, weekly hospitalizations were 0.28 lower for the QuietCare® facility than for the control facility; however, this difference was not statistically significant (Table 3).

**TABLE 3:** Time Series Analyses

Variable	Coefficient	*p*-value
	Falls	
Intercept	3.54	<0.0001
Week	−0.03	0.019
Facility ID	−1.08	0.008
	Hospitalizations	
Intercept	1.69	<0.0001
Week	−0.01	0.08
Facility ID	−0.28	0.22

**Figure 2:**
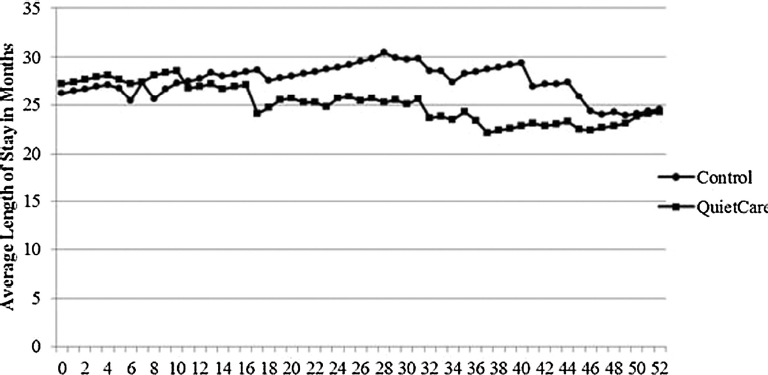
Average length of stay.

For each week of the study, baseline (0) to week 52, there were no statistically significant differences in the average length of stay between the two facilities. However, average lengths of stay at the QuietCare® facility began to decline starting at week 10 (Figure 2). Investigations revealed that there were more respite residents in the QuietCare® facility than in the control facility (7 compared to 1). This and other efforts at the QuietCare® facility to increase its occupancy resulted in a decreasing average length of stay during the study period.

## DISCUSSION

Studies have shown that remote monitoring systems can be used to detect behavioral patterns. Identifiable events include sleep behavior, changing clothes, bathroom/toilet use, leaving/returning home, and meal preparation, which constitute the majority of the activities of daily living that are used in functional assessments performed by healthcare professionals (Barger et al, 2005). This study provides evidence of direct benefits to both residents and assisted living facilities for the use of QuietCare® facility, a passive monitoring system. While the QuietCare® facility had a higher percentage of patients in care level 1 at baseline, a greater percentage of residents at the QuietCare® facility were in higher care levels than the control facility by the end of the study (24% versus 17%). This study demonstrated that these residents could be maintained in the QuietCare® facility without having to move to more restrictive settings, such as nursing homes. This led to greater consistency in care level at the QuietCare® facility. Despite, there being more residents at higher care levels during the study period, there were fewer falls (0.48 versus 1.33; *p* = 0.0001) and a trend toward fewer hospitalizations (0.92 versus 1.08; ns) per week in the QuietCare® facility. In addition, the QuietCare® facility had a higher resident retention rate, with 63 % of residents remaining at the facility throughout the study period, compared with 49% at the control facility.

This study did not demonstrate longer lengths of stay at the QuietCare® facility compared with the control facility. This was primarily due to two factors: first, the QuietCare® facility had more respite residents than the control facility (7 compared to 1) over the study timeframe, and second, the QuietCare® facility increased its occupancy over time. With more new residents coming into the facility during the study window, the average length of stay decreased.

Successful assisted living facilities optimize available resources to achieve a higher standard of care for their residents. By providing information that may reduce falls and hospitalizations, the QuietCare® system may help seniors stay independent for longer periods. This study demonstrated that although residents at the QuietCare® site declined at a faster rate than residents at the control facility (data not shown), they were able to age-in-place rather than being moved to a nursing home or other more restrictive care setting.

In addition, the QuietCare® system may actually help improve revenue of assisted living communities. Financial information from the two facilities revealed that while revenue declined and expenses increased at the control facility, both revenue and expenses increased at the QuietCare® facility. However, revenue increased faster than expenses (data not shown). These results are similar to the earlier studies (GE QuietCare, 2011) that illustrated the positive impact of QuietCare® on facility revenue. One study showed a need for higher levels of care and associated service levels that resulted in an average monthly service increase of $107 per resident in revenue. These service levels help residents to stay where they are even as they increase their need for care: in instances where more acute care is needed (such as dementia), residents can be transferred to higher care areas proactively before more serious risks emerge.

Limitations of this study included the fact that data were collected at only two facilities: one utilizing the GE QuietCare® passive monitoring system, and the other not, which led to a relatively small sample size. Also, while these two facilities were similar in terms of baseline demographics, there may have been differences in adherence of policies, such as care level assessment as well as differences in staffing that were not captured and may have had some impact on the outcomes of the study. These factors would be difficult to control for when comparing facilities.

## CONCLUSIONS

This study provides evidence of direct benefits to both resident and facility for the use of QuietCare®, a passive monitoring system. There was a significant reduction in the number of falls, as well as a general facility performance improvement measured by care level consistency and higher resident retention rates. Aging in place is important to seniors, and monitoring systems may help improve overall quality of life and independence.

## ABOUT THE AUTHORS

**Dr. Pankaj Patel** is Senior Director and Head of Global Evidence Generation in the Health Economics team at GE Healthcare. Dr. Patel has authored over 75 publications & scientific conference presentations and is also a guest lecturer at the University of Illinois College of Pharmacy. **Dr. Candace Gunnarsson** is the President of S^2^ Statistical Solutions, located in Cincinnati, Ohio that provides statistical expertise, outcomes research, economic modeling and customized software solutions for pharmaceutical and medical device companies in and outside the United States. Prior to founding S^2^ Dr. Gunnarsson was a professor where she taught statistics courses for over ten years in the fields of education, psychology, and business at Xavier University and University of Cincinnati.
